# Surgery-related factors associated with early postoperative kinesiophobia in geriatric hip fracture patients: a risk stratification model

**DOI:** 10.3389/fmed.2026.1876658

**Published:** 2026-09-17

**Authors:** Peijia Jing, Han-chi Liu, Jia-luo Cai, Huaitao Zhu, Shaxin Liu

**Affiliations:** 1 Rehabilitation Medicine Center, West China Hospital, Sichuan University, Chengdu, China; 2 Department of Rehabilitation, Langzhong People’s Hospital, Nanchong, China; 3 Department of Rehabilitation, University-Town Hospital of Chongqing Medical University, Chongqing, China; 4 Rehabilitation Department, The Second People’s Hospital of Neijiang, Neijiang, China

**Keywords:** hip fracture, influencing factors, kinesiophobia, risk stratification model, surgical predictors

## Abstract

**Objective:**

To explore the current situation and surgery-related influencing factors of kinesiophobia in geriatric hip fracture (GHF) patients in the early postoperative period.

**Methods:**

This study included 209 elderly hip fracture patients who underwent surgical treatment between March 2023 and March 2025. Kinesiophobia was assessed using the Tampa Scale for Kinesiophobia (TSK), while pain levels were measured using the Visual Analogue Scale (VAS). The Harris Hip Score (HHS) was used to evaluate hip function. Operative time and estimated blood loss were recorded from surgical records. Logistic regression analysis was performed to identify factors influencing kinesiophobia, and a risk model was constructed and evaluated.

**Results:**

The incidence of kinesiophobia was 34.0%. Multivariate logistic regression analysis identified operative time (per 10 min increase: OR = 1.75, 95% CI: 1.32–2.31), estimated blood loss (per 10 mL increase: OR = 1.18, 95% CI: 1.09–1.29), VAS score (OR = 3.57, 95% CI: 2.01–6.33), HHS score (OR = 0.83, 95% CI: 0.75–0.91), and age (OR = 1.14, 95% CI: 1.05–1.25) as independent factors associated with kinesiophobia. The risk model demonstrated good discriminatory ability with an AUC of 0.89 (95% CI: 0.85–0.94) and satisfactory calibration (Hosmer-Lemeshow test *P* = 0.836).

**Conclusion:**

Kinesiophobia in GHF patients at the early postoperative stage is closely related to surgical invasiveness (operative time and blood loss), pain, advanced age, and poor hip joint function. The risk model constructed based on these concurrently assessed factors may aid in the early identification of patients with high levels of early postoperative fear and inform early monitoring strategies. However, given the cross-sectional, single-time-point design of this study, longitudinal studies are needed to determine whether this early fear persists and affects long-term functional recovery.

## Introduction

Hip fracture (HF) is one of the most common severe traumatic injuries in the elderly, with its incidence continuing to rise as the population ages (1). Globally, an estimated 14.2 million incident hip fractures occurred in 2019, with an age-standardized incidence rate of 182 per 100,000 population, posing a substantial burden on healthcare systems worldwide (2). Age-standardized incidence rates varied considerably by age and sex, with higher rates in older adults and females, and the male-to-female ratio among those aged over 55 years increased from 0.577 in 1990 to 0.612 in 2019 (3). GHF mainly includes femoral neck fractures and intertrochanteric fractures, typically caused by low-energy trauma such as falls (4). While both fracture types share similar injury mechanisms, intertrochanteric fractures are generally associated with greater blood loss and longer operative times due to the need for more extensive surgical exposure and fixation (4). Beyond significantly impairing quality of life, GHF is also associated with high rates of disability and mortality, with reported short-term mortality reaching 14.2% within 3 months and cumulative mortality approaching 34.2% at 2 years post-fracture (5). Surgical treatment remains the primary intervention, as it effectively restores bone stability and facilitates early mobilization (6). Early surgical intervention, ideally within 48 h of admission, has been consistently associated with reduced mortality, shorter hospital stays, and improved functional outcomes, making timely surgery a cornerstone of contemporary hip fracture management (6). However, postoperative recovery is particularly challenging in elderly patients due to age-related physiological decline. The aging process is characterized by diminished physiological reserve, reduced muscle mass (sarcopenia), impaired neuromuscular coordination, and increased prevalence of multimorbidity, all of which compromise the ability to tolerate surgical stress and engage in postoperative rehabilitation (7). Active functional exercise in the early postoperative period is critical for fracture healing and recovery. However, this process is frequently impeded by patients’ fear of pain and activity avoidance, a condition referred to as “kinesiophobia” (8).

Kinesiophobia is defined as an excessive, irrational fear of pain or re-injury triggered by movement, and is particularly common among postoperative orthopaedic patients (9). Originally described by Kori and colleagues in the context of chronic pain syndromes, the concept of kinesiophobia has since been extensively studied across diverse musculoskeletal conditions, including low back pain, neck pain, and osteoarthritis (9). In the postoperative orthopaedic setting, kinesiophobia manifests as a persistent reluctance to engage in physical activity despite medical clearance, driven by catastrophic misinterpretation of bodily sensations during movement. This fear leads to reduced activity levels, which in turn promotes muscle atrophy, joint stiffness, and delayed recovery (9, 10). The vicious cycle of “pain–fear–avoidance” has been well characterized in the pain literature: pain triggers fear, fear drives avoidance behavior, avoidance leads to physical deconditioning and functional decline, and deconditioning exacerbates pain perception, thereby reinforcing the cycle (8, 9). In elderly hip fracture patients, this cycle is particularly detrimental, as even short periods of immobilization can result in rapid muscle loss and irreversible functional decline (10). Although kinesiophobia affects over 50% of elderly fracture patients (11, 12), most prior research has concentrated on the mid- to late postoperative phase, leaving the early postoperative period—a critical window for recovery—relatively underexplored (13, 14). Specifically, the majority of existing studies have focused on patients with total knee or hip arthroplasty for osteoarthritis, rather than those with acute traumatic fractures, and have typically assessed kinesiophobia at 3–12 months post-surgery (11–13). This focus on chronic rather than acute phases has left a significant gap in our understanding of how kinesiophobia develops in the immediate postoperative days, when rehabilitation efforts are most impactful (14).

From a surgical standpoint, several intraoperative and postoperative factors may contribute to kinesiophobia. Prolonged operative time and greater blood loss reflect increased surgical invasiveness, potentially leading to heightened postoperative pain, delayed recovery, and exacerbated fear of movement (15, 16). Longer operative time is often associated with more complex fracture patterns, greater soft tissue dissection, and increased intraoperative blood loss, all of which contribute to a more pronounced systemic inflammatory response and greater postoperative pain intensity (15). Similarly, substantial intraoperative hemorrhage may necessitate transfusion, which has been independently linked to postoperative delirium, prolonged hospitalization, and poorer functional recovery—factors that may indirectly heighten patients’ perception of vulnerability and fear of movement (16). Moreover, poor early hip function and severe pain have been associated with reduced rehabilitation compliance (17). Pain, in particular, is thought to act as a central driver of kinesiophobia through its effects on fear-conditioning circuits in the brain, especially the amygdala and anterior cingulate cortex, which process the affective-motivational dimension of pain and generate avoidance responses (17). Despite these plausible links, the specific surgery-related predictors of early postoperative kinesiophobia in GHF patients have not been systematically investigated. In particular, no study to date has integrated intraoperative parameters (operative time and blood loss) with early postoperative clinical assessments (pain intensity and functional status) in a single predictive model for kinesiophobia in this vulnerable population. Therefore, this study aims to identify surgery-related factors—including operative time, estimated blood loss, pain intensity, hip function, and complications—that are concurrently associated with early postoperative kinesiophobia in GHF patients, and to develop a risk stratification nomogram based on these factors to guide early perioperative interventions. Such a tool would enable clinicians to identify patients with high levels of early postoperative fear and implement personalized interventions—including enhanced analgesia, early psychological support, and graded mobilization protocols—to reduce fear and facilitate early rehabilitation. However, it should be noted that the cross-sectional, single-time-point design of this study precludes prediction of persistent kinesiophobia or long-term functional outcomes; future longitudinal studies are needed to determine the clinical trajectory of these early findings.

## Materials and methods

### Study participants

This study used a mixed investigative research design, combining retrospective data extraction from electronic medical records with prospective collection of questionnaire-based assessments, to consecutively include elderly hip fracture patients who were admitted to our orthopaedic department between March 2023 and March 2025 and underwent surgical treatment. After a rigorous inclusion and exclusion procedure, data from 209 patients were included for analysis. A total of 293 consecutive patients were initially screened, of whom 84 were excluded; the detailed screening process and reasons for exclusion are presented in Figure 1. The inclusion criteria were as follows: (1) age ≥60 years; (2) hip fracture diagnosed by imaging; (3) received surgical treatment, including hip arthroplasty (hemiarthroplasty or total hip arthroplasty) and internal fixation (intramedullary nailing or dynamic hip screw); (4) postoperative consciousness and ability to cooperate in completing the questionnaire; (5) complete clinical data. The exclusion criteria were as follows: (1) patients with a combination of severe cognitive impairment, mental illness, and inability to communicate normally; (2) the presence of severe cardiac, hepatic, renal, and other important organ failure; (3) a previous history of hip fracture or hip surgery; (4) pathological fractures; (5) those who were transferred to the hospital due to deterioration of the condition during the postoperative hospitalisation period or died; and (6) incomplete clinical data, including missing surgical records or incomplete questionnaire assessments. The study strictly adhered to all principles of the Declaration of Helsinki. The study was approved by the Hospital Ethics Committee. Written informed consent was obtained from all participants or their legal guardians prior to enrollment. Consent forms were signed on the day of admission, after a thorough explanation of the study purpose, procedures, and potential risks had been provided.

**Figure 1:**
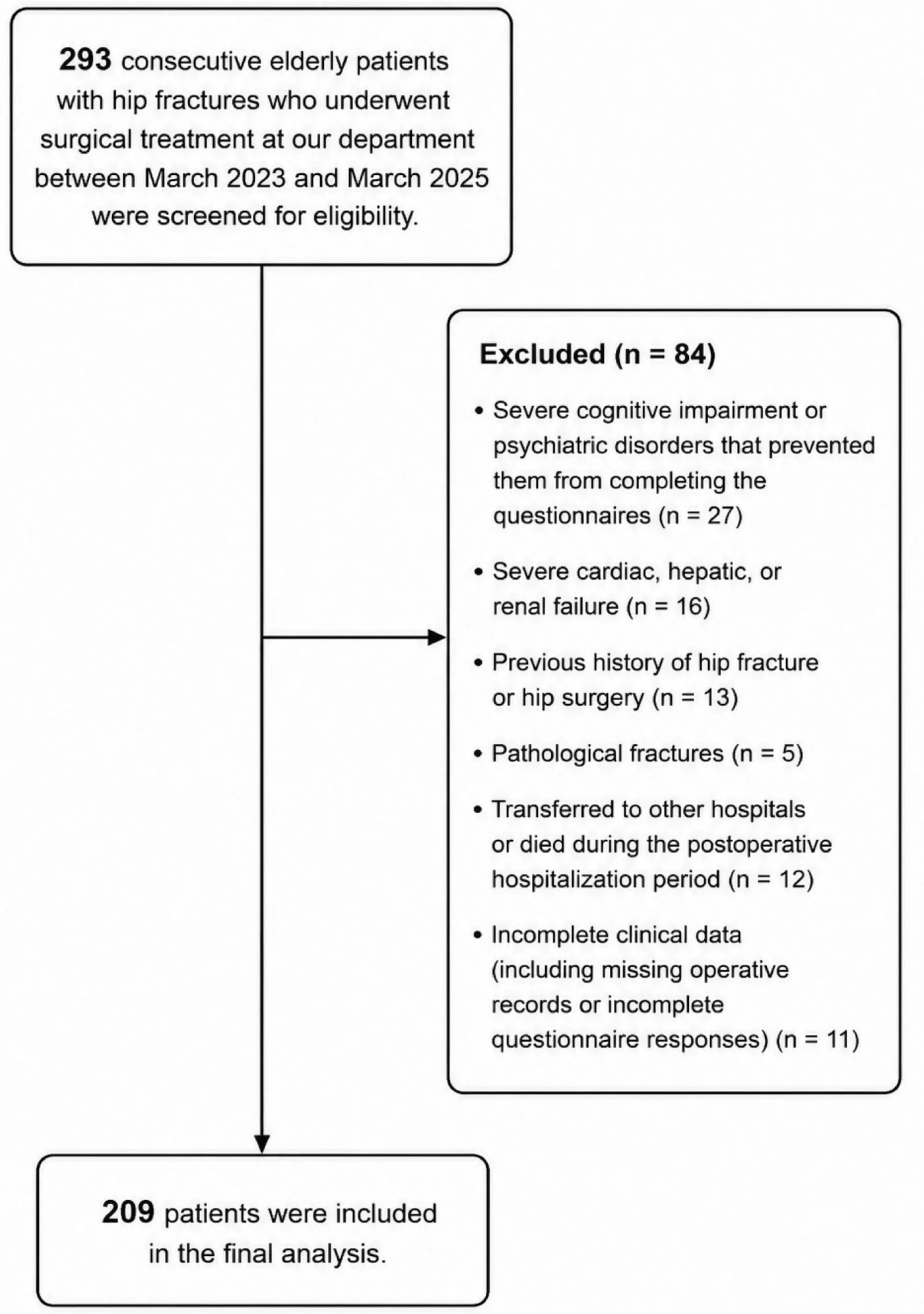
Flowchart of patient screening and enrollment.

### Participant characteristics

A total of 209 GHF patients were included, comprising 71 (34.0%) in the kinesiophobia group and 138 (66.0%) in the non-kinesiophobia group. As shown in Table 1, patients in the kinesiophobia group were significantly older than those in the non-kinesiophobia group (median 76.00 vs. 73.00 years, *Z* = −3.52, *P* < 0.001). Regarding comorbidities, a higher prevalence of diabetes mellitus was observed in the kinesiophobia group (28.17% vs. 15.22%, *P* = 0.026). No significant differences were found between the two groups in terms of gender, BMI, education, marital status, place of residence, hypertension, hyperlipidemia, or coronary heart disease (all *P* > 0.05).

**Table 1:** Baseline demographic and clinical characteristics of geriatric hip fracture patients (*N* = 209).

Variables	Total (*n* = 209)	Non-kinesiophobia (*n* = 138)	Kinesiophobia group (*n* = 71)	Statistic	*P*
Age, M (Q₁, Q₃)	74.00 (70.00, 77.00)	73.00 (69.00, 76.00)	76.00 (71.50, 78.00)	*Z* = −3.52	<0.001
BMI, M (Q₁, Q₃)	21.51 (20.06, 23.23)	21.51 (20.15, 22.98)	21.45 (19.85, 23.71)	*Z* = −0.54	0.591
Gender, *n* (%)				*χ*^2^ = 0.09	0.759
Female	97 (46.41)	63 (45.65)	34 (47.89)		
Male	112 (53.59)	75 (54.35)	37 (52.11)		
Educational background, *n* (%)				*χ*^2^ = 1.34	0.248
Above high school	79 (37.80)	56 (40.58)	23 (32.39)		
High school and below	130 (62.20)	82 (59.42)	48 (67.61)		
Marital status, *n* (%)				*χ*^2^ = 1.52	0.218
Married	182 (87.08)	123 (89.13)	59 (83.10)		
Unmarried/divorced/widowed	27 (12.92)	15 (10.87)	12 (16.90)		
Place of residence, *n* (%)				*χ*^2^ = 1.11	0.292
City	90 (43.06)	63 (45.65)	27 (38.03)		
Rural area	119 (56.94)	75 (54.35)	44 (61.97)		
Comorbidities, *n* (%)					
Hypertension	80 (38.28)	49 (35.51)	31 (43.66)	*χ*^2^ = 1.32	0.251
Hyperlipidemia	72 (34.45)	45 (32.61)	27 (38.03)	*χ*^2^ = 0.61	0.435
Diabetes mellitus	41 (19.62)	21 (15.22)	20 (28.17)	*χ*^2^ = 4.99	0.026
Coronary heart disease	37 (17.70)	22 (15.94)	15 (21.13)	*χ*^2^ = 0.86	0.352

Table 2 presents the comparison of surgery-related characteristics between the two groups. Patients in the kinesiophobia group had significantly longer operative times (median 92.00 vs. 79.00 min, *Z* = −5.69, *P* < 0.001) and higher estimated blood loss (median 319.10 vs. 286.20 mL, *Z* = −4.88, *P* < 0.001). In terms of postoperative recovery, the kinesiophobia group showed lower HHS scores (median 68.00 vs. 72.00, *Z* = −4.73, *P* < 0.001) and higher VAS pain scores (median 3.00 vs. 3.00, *Z* = −5.47, *P* < 0.001). Additionally, the incidence of postoperative complications was significantly higher in the kinesiophobia group (25.35% vs. 12.32%, *χ*^2^ = 5.71, *P* = 0.017). No significant differences were observed in fracture type, anesthesia method, or surgical procedure between the two groups (all *P* > 0.05).

**Table 2:** Surgery-related characteristics of geriatric hip fracture patients (*N* = 209).

Variables	Total (*n* = 209)	Non-kinesiophobia (*n* = 138)	Kinesiophobia group (*n* = 71)	Statistic	*P*
Fracture type, *n* (%)				*χ*^2^ = 0.96	0.327
Trochanteric fracture	73 (34.93)	45 (32.61)	28 (39.44)		
Femoral neck fracture	136 (65.07)	93 (67.39)	43 (60.56)		
Anesthesia method, *n* (%)				*χ*^2^ = 0.83	0.363
Non-general anesthesia	51 (24.40)	31 (22.46)	20 (28.17)		
General anesthesia	158 (75.60)	107 (77.54)	51 (71.83)		
Surgical method, *n* (%)				*χ*^2^ = 0.96	0.326
Hip arthroplasty	153 (73.21)	104 (75.36)	49 (69.01)		
Internal fixation	56 (26.79)	34 (24.64)	22 (30.99)		
Operative time (min), M (Q₁, Q₃)	82.00 (74.00, 93.00)	79.00 (71.00, 86.00)	92.00 (81.00, 103.00)	*Z* = −5.69	<0.001
Estimated blood loss (mL), M (Q₁, Q₃)	299.00 (260.80, 333.30)	286.20 (251.47, 316.62)	319.10 (289.00, 362.40)	*Z* = −4.88	<0.001
HHS score, M (Q₁, Q₃)	71.00 (67.00, 75.00)	72.00 (69.00, 75.00)	68.00 (66.00, 72.50)	*Z* = −4.73	<0.001
VAS score, M (Q₁, Q₃)	3.00 (2.00, 3.00)	3.00 (2.00, 3.00)	3.00 (3.00, 4.00)	*Z* = −5.47	<0.001
Postoperative complications, *n* (%)	35 (16.75)	17 (12.32)	18 (25.35)	*χ*^2^ = 5.71	0.017

### Sample size calculation

This was a single-center retrospective observational study, and no *a priori* power calculation was performed for sample size determination. Instead, the required sample size was estimated based on the rule of thumb for logistic regression analysis. According to the widely adopted recommendation of at least 10 events per predictor variable (EPV) for reliable model estimation (18), and considering that we planned to include up to 5 independent predictors in the multivariate mode, a minimum of 50 outcome events (i.e., patients with kinesiophobia) would be required. In the present study, a total of 71 patients (34.0%) developed early postoperative kinesiophobia, which corresponds to an EPV of approximately 14.2 (71/5 = 14.2), exceeding the recommended minimum threshold. This sample size ensured adequate statistical power and model stability for the planned multivariate logistic regression analyses.

### Questionnaires

Patients’ level of kinesiophobia was assessed using the Tampa Scale for Kinesiophobia (TSK), which consists of 17 items rated on a 4-point scale (1 = Strongly Disagree, 4 = Strongly Agree), with a total score of 17–68, with higher scores indicating a greater level of fear of exercise (19). The Chinese version of TSK has been proven to have high application value among the Chinese population (20). In this study, the Cronbach's *α* coefficient of the TSK scale was 0.792. A TSK total score ≥ 38 was used to define the presence of kinesiophobia, consistent with previous studies in orthopaedic populations and related patient cohorts (21, 22). Specifically, this cutoff was adopted in a recent study of hip fracture patients by Xu and Chen (21) and was also used in the context of anterior cruciate ligament injury by Grinberg et al. (22), supporting its utility across different musculoskeletal conditions.

The Visual Analogue Scale (VAS) was used to assess the patient's postoperative pain level, and a 10 cm long straight line was marked with a score of ‘0’ (no pain) and ‘10’ (severe pain) at each end. The patients were asked to mark the corresponding locations on the line according to their pain perception (23).

The Harris Hip Score (HHS) was used to evaluate the hip joint function of patients (24). HHS encompasses four dimensions: pain (44 points), function (47 points), deformity (4 points), and joint range of motion (5 points). The total score of HHS is 100 points. The higher the score, the better the hip joint function. For the functional items (e.g., using public transportation, putting on shoes and socks, climbing stairs), patients were asked to report their pre-fracture (pre-admission) functional level, as these activities could not be directly observed during the early inpatient period. For hip range of motion, assessment was performed within the limits allowed by postoperative restrictions (e.g., hip flexion ≤ 90° in arthroplasty patients), and the scores were documented accordingly. The pain dimension of the HHS was scored based on the patient's current pain level at the time of assessment, which was directly applicable to the inpatient setting.

### Procedure

All questionnaire and scale assessments (TSK, VAS, and HHS) were completed by trained researchers during face-to-face interviews with patients on postoperative day 5 (range: day 4 to day 6). It is important to clarify that these specific psychometric scales are not part of our hospital's routine clinical protocol. Therefore, for the purpose of this study, we prospectively collected these data specifically for research purposes. The assessments were administered in a fixed order for all participants: the VAS was completed first, followed by the HHS, and finally the TSK. This order was chosen to minimize the potential influence of fatigue on the more comprehensive TSK assessment. The questionnaires were administered by two trained research assistants (not the treating surgeons or nurses) who were independent of the patients’ clinical care and blinded to the patients’ surgical details at the time of assessment. The following surgery-related variables were retrospectively extracted from the electronic medical record system by trained research staff blinded to the patients’ questionnaire responses: age, gender, body mass index (BMI), education, marital status, place of residence, chronic diseases (including hypertension, hyperlipidemia, diabetes mellitus, and coronary heart disease), fracture type, anesthesia modality, surgical modality, operative time (minutes), estimated intraoperative blood loss (mL), postoperative complications (including pneumonia, urinary tract infection, deep vein thrombosis, pressure ulcers, delirium, wound complications (such as dehiscence, infection, or hematoma), and new-onset cardiovascular events (e.g., heart failure, arrhythmia, or myocardial infarction)), and postoperative hospital stay. Thus, while the clinical and surgical data were obtained from historical records, the primary outcome (kinesiophobia) and key clinical correlates (pain and function) were collected prospectively.

### Statistical analysis

SPSS26.0 was used for statistical analysis of the data in this study. Due to the exclusion of patients with incomplete medical records during the enrollment process, all included cases had complete data for all study variables. Thus, a complete-case analysis was performed without the need for additional imputation. Among the continuous variables, those conforming to the normal distribution were described as the mean (standard deviation, SD), and the independent sample t-test was used for comparison between groups. Those not conforming to the normal distribution were described as the median (interquartile ranges, IQR), and the Mann–Whitney U test was used for comparison between groups. Categorical variables were described as frequencies (percentages) and comparisons between groups were made using the chi-square test. The influencing factors of early postoperative kinesiophobia were screened through univariate Logistic regression analysis, and the odds ratio (OR) and 95% confidence interval (95% CI) were calculated. A forward stepwise selection procedure (entry criterion: *P* < 0.05; removal criterion: *P* > 0.10) was then applied to identify independent predictors. Multicollinearity among independent variables in the multivariate logistic regression model was assessed using the variance inflation factor (VIF), with a VIF value > 10 considered indicative of significant collinearity. Based on the results of multivariate analysis, a nomogram risk model was constructed. The model's discriminative ability was evaluated by the receiver operating characteristic (ROC) curve and the area under the curve (AUC). The consistency between the predicted probability and the actual probability was evaluated by the calibration curve and the Hosmer-Lemeshow test. The clinical net benefit was evaluated by decision curve analysis (DCA). The accuracy rate, sensitivity, specificity, positive predictive value (PPV) and negative predictive value (NPV) of the model were calculated using the confusion matrix to determine the optimal cut-off value. Internal validation was performed using 500 bootstrap resamples to correct for overfitting and to estimate the optimism of the model's performance. The bootstrap-corrected AUC and calibration slope were calculated as the difference between the apparent performance and the average optimism derived from the resampling procedure. Calibration was assessed by plotting the predicted probabilities against the observed outcomes, with a calibration slope of 1.0 and an intercept of 0 indicating perfect agreement. The test level *α*=0.05, and *P* < 0.05 was considered statistically significant.

## Results

### Univariate logistic regression analysis

Univariate analysis identified several factors significantly associated with postoperative kinesiophobia (Table 3). Pain intensity (VAS score) showed the strongest association (OR = 3.30, 95% CI: 2.07–5.27, *P* < 0.001), followed by operative time (per 10 min increase: OR = 1.92, 95% CI: 1.51–2.45, *P* < 0.001) and estimated blood loss (per 10 mL increase: OR = 1.18, 95% CI: 1.10–1.26, *P* < 0.001). Other significant predictors included age (OR = 1.11, 95% CI: 1.04–1.19, *P* < 0.001), lower HHS score (OR = 0.85, 95% CI: 0.79–0.91, *P* < 0.001), the presence of diabetes (OR = 2.18, 95% CI: 1.09–4.38, *P* = 0.028), and postoperative complications (OR = 2.42, 95% CI: 1.16–5.05, *P* = 0.019). BMI, gender, education level, marital status, residence, fracture type, anesthesia method, and surgical method were not significant predictors (all *P* > 0.05).

**Table 3:** Univariate logistic regression analysis.

Variables	*β*	S.E	*Z*	*P*	OR (95% CI)
Age	0.11	0.03	3.30	<0.001	1.11 (1.04∼1.19)
BMI	0.06	0.06	1.02	0.308	1.06 (0.94∼1.20)
Gender (Male)	−0.09	0.29	−0.31	0.759	0.91 (0.52∼1.62)
Educational background (High school and below)	0.35	0.31	1.15	0.249	1.43 (0.78∼2.60)
Marital status (Unmarried/divorced/widowed)	0.51	0.42	1.22	0.222	1.67 (0.73∼3.79)
Place of residence (Rural area)	0.31	0.30	1.05	0.292	1.37 (0.76∼2.46)
Hypertension	0.34	0.30	1.15	0.251	1.41 (0.78∼2.53)
Hyperlipidemia	0.24	0.30	0.78	0.435	1.27 (0.70∼2.30)
Diabetes	0.78	0.35	2.20	0.028	2.18 (1.09∼4.38)
Coronary heart disease	0.35	0.37	0.93	0.354	1.41 (0.68∼2.93)
Fracture type (Femoral neck fracture)	−0.30	0.30	−0.98	0.327	0.74 (0.41∼1.35)
Anesthesia method (General anesthesia)	−0.30	0.33	−0.91	0.364	0.74 (0.38∼1.42)
Surgical method (Internal fixation)	0.32	0.32	0.98	0.327	1.37 (0.73∼2.59)
Operative time (per 10 min increase)	0.65	0.12	5.26	<0.001	1.92 (1.51∼2.45)
Estimated blood loss (per 10 mL increase)	0.16	0.03	4.86	<0.001	1.18 (1.10∼1.26)
HHS score	−0.16	0.04	−4.56	<0.001	0.85 (0.79∼0.91)
VAS score	1.20	0.24	5.02	<0.001	3.30 (2.07∼5.27)
Postoperative complications	0.88	0.38	2.35	0.019	2.42 (1.16∼5.05)

### Multivariate logistic regression analysis

Multivariate analysis (Table 4) was performed incorporating variables with *P* < 0.05 from the univariate analysis. This analysis confirmed that operative time (per 10 min increase: OR = 1.75, 95% CI: 1.32–2.31, *P* < 0.001), estimated blood loss (per 10 mL increase: OR = 1.18, 95% CI: 1.09–1.29, *P* < 0.001), higher VAS score (OR = 3.57, 95% CI: 2.01–6.33, *P* < 0.001), lower HHS score (OR = 0.83, 95% CI: 0.75–0.91, *P* < 0.001), and older age (OR = 1.14, 95% CI: 1.05–1.25, *P* = 0.002) were independent predictors of early postoperative kinesiophobia in GHF patients. A nomogram incorporating these five independent predictors was constructed to provide a individualized risk assessment tool for clinicians (Figure 2). Collinearity diagnostics using VIF revealed that all VIF values for the five independent predictors were below 2.0 (range: 1.12–1.89), indicating no significant multicollinearity among the variables in the final multivariate model.

**Table 4:** Multivariate logistic regression analysis.

Variables	*β*	S.E	*Z*	*P*	OR (95% CI)
Intercept	−10.75	4.68	−2.30	0.022	0.00 (0.00∼0.21)
VAS score	1.27	0.29	4.35	<0.001	3.57 (2.01∼6.33)
HHS score	−0.19	0.05	−3.84	<0.001	0.83 (0.75∼0.91)
Age	0.13	0.04	3.09	0.002	1.14 (1.05∼1.25)
Operative time (per 10 min increase)	0.56	0.14	3.93	<0.001	1.75 (1.32∼2.31)
Estimated blood loss (per 10 mL increase)	0.17	0.04	3.87	<0.001	1.18 (1.09∼1.29)

**Figure 2:**
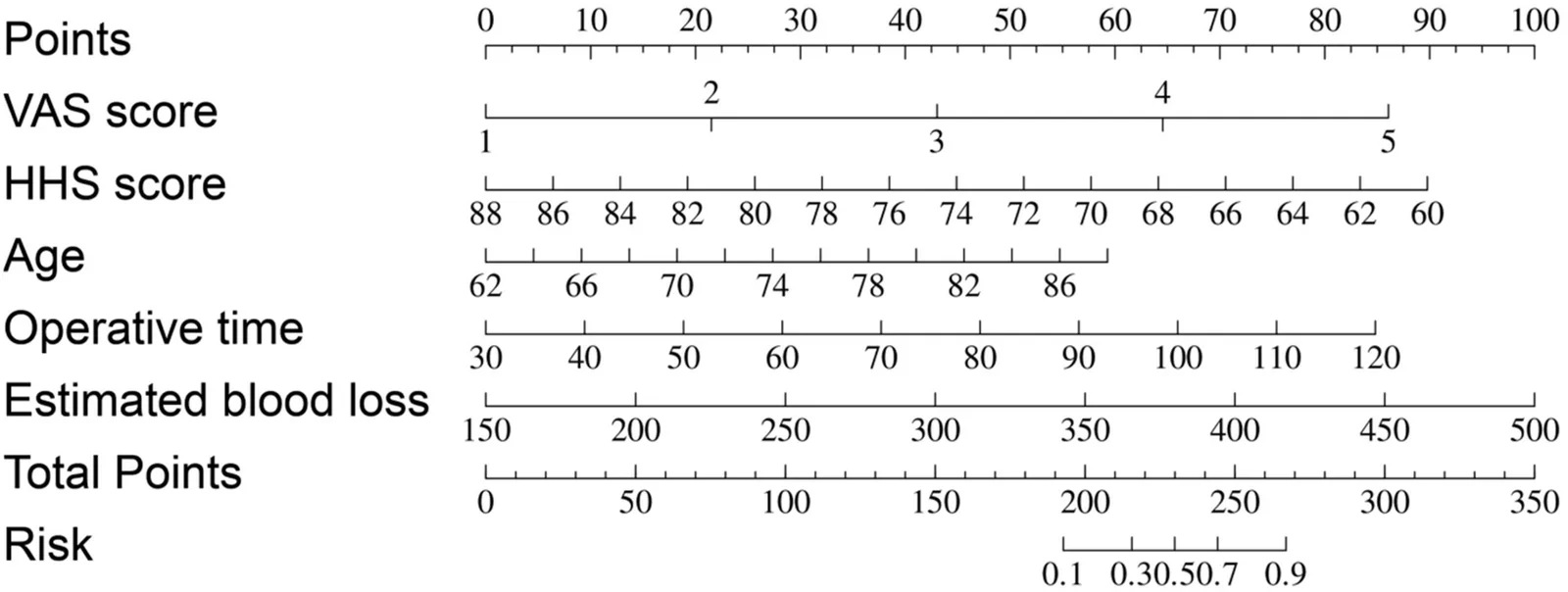
Nomogram prediction model.

### Performance of the nomogram risk model

The discriminative performance of the nomogram was evaluated using ROC curve analysis. The model demonstrated excellent discriminatory ability with an AUC of 0.89 (95% CI: 0.85–0.94) (Figure 3). Calibration curve analysis showed good agreement between the predicted probabilities and the observed outcomes, and the Hosmer-Lemeshow test yielded a *P*-value of 0.836, indicating satisfactory calibration performance (Figure 4). DCA revealed that the model provided a positive net clinical benefit across a wide range of threshold probabilities (0.05–1.0), suggesting its clinical utility for guiding perioperative interventions (Figure 5).

**Figure 3:**
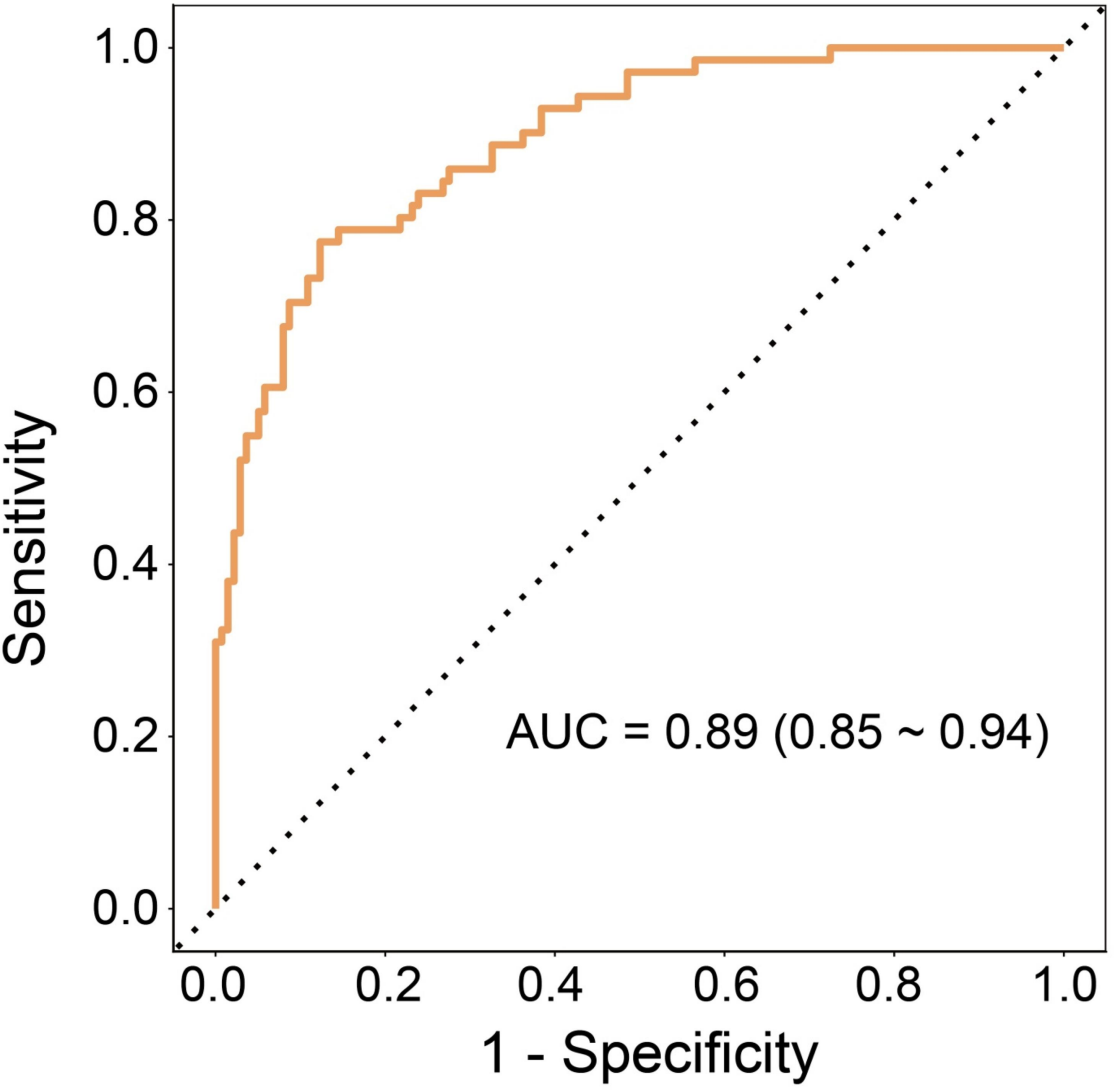
ROC curve analysis.

**Figure 4:**
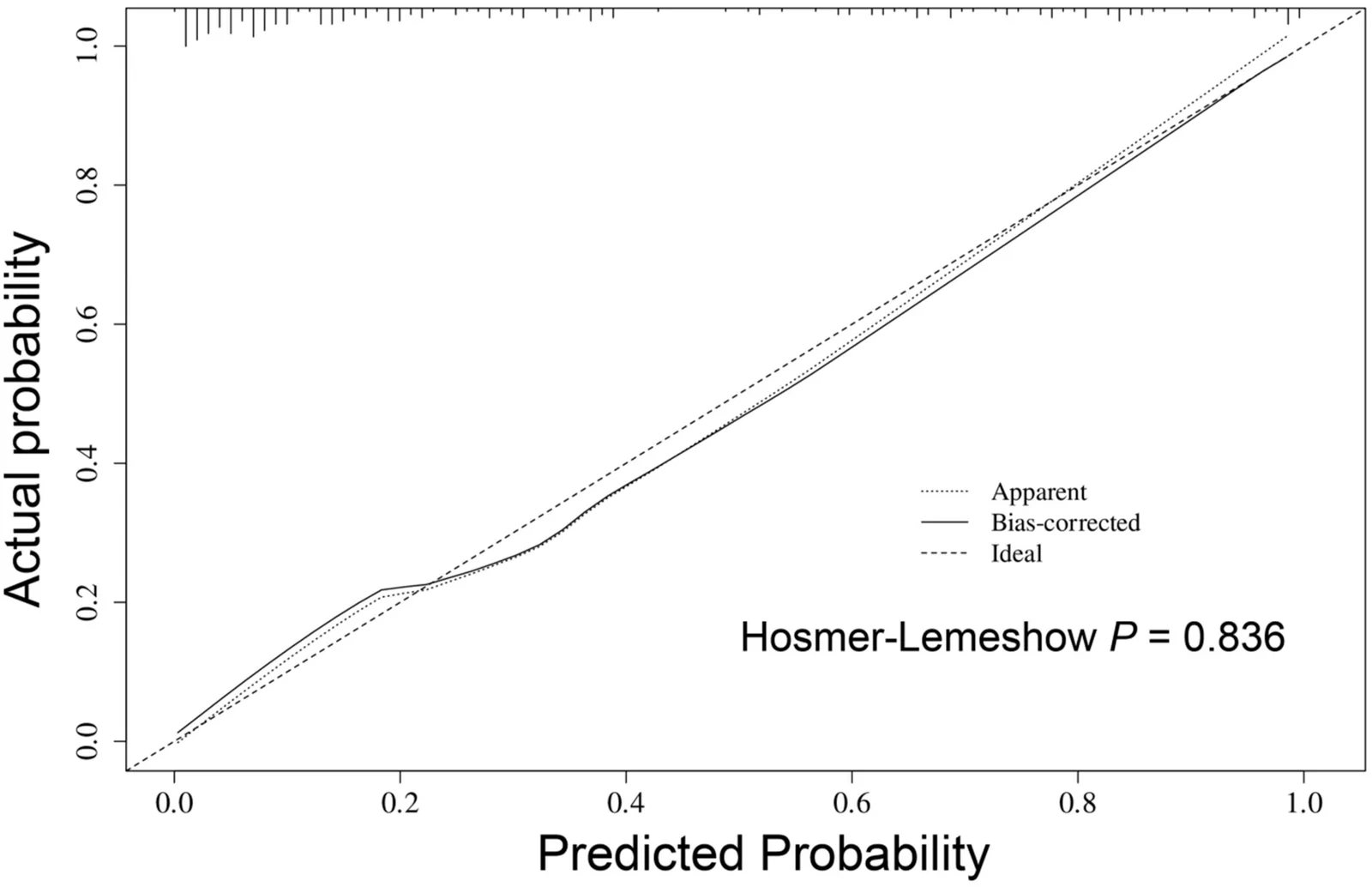
Calibration curve analysis.

**Figure 5:**
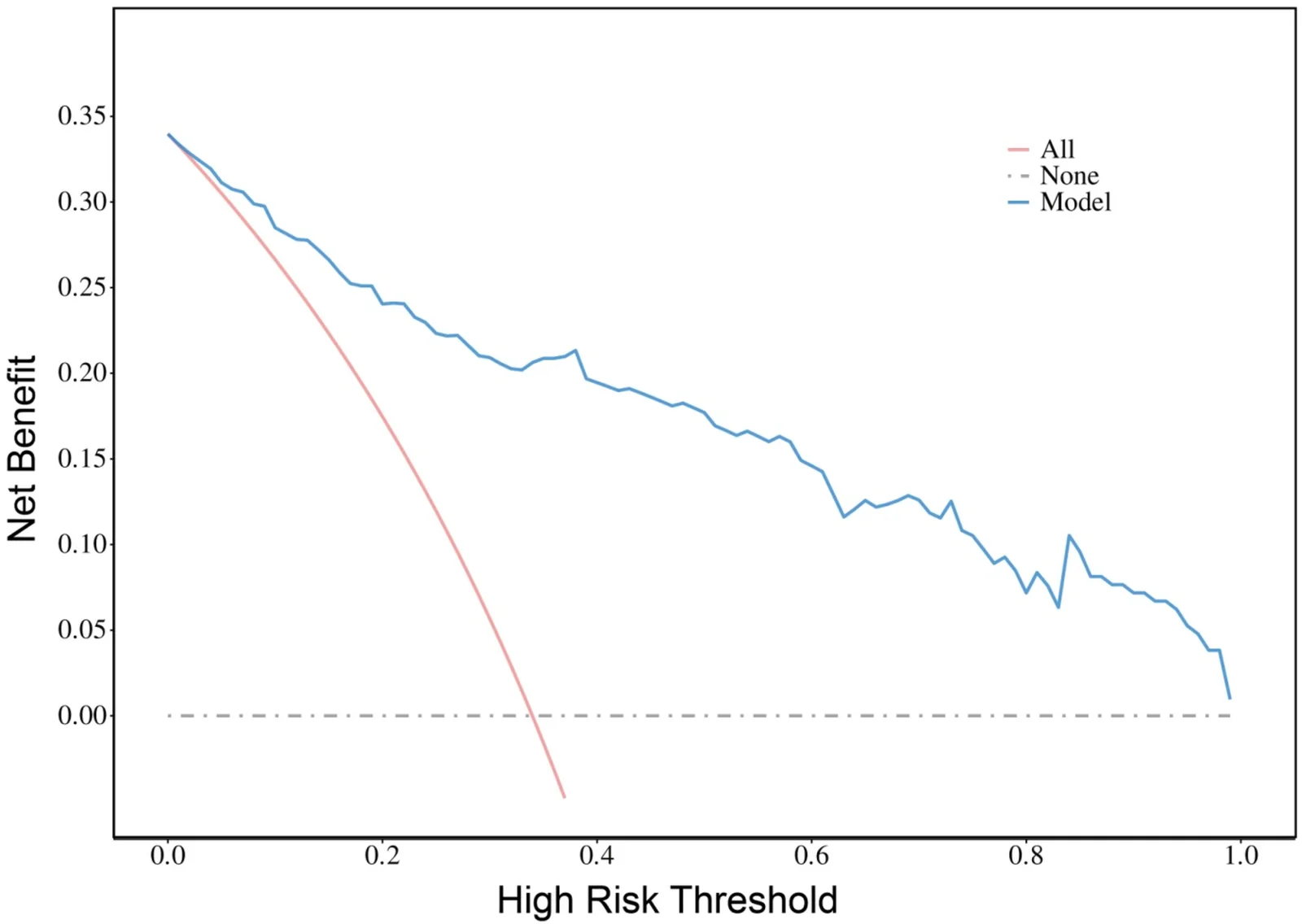
Clinical decision curve.

### Confusion matrix analysis

As shown in Table 5, the nomogram achieved an overall accuracy of 0.84 (95% CI: 0.79–0.89). Based on the Youden index, the optimal cut-off probability for predicting early postoperative kinesiophobia was 0.38. Patients with a predicted probability ≥ 0.38 were classified as high-risk, while those with a probability < 0.38 were classified as low-risk. At this threshold, the model achieved a sensitivity of 0.77 (95% CI: 0.68–0.87) and a specificity of 0.88 (95% CI: 0.82–0.93), as detailed in Table 5. This cut-off value can serve as a practical bedside reference for clinicians to identify high-risk patients in the early postoperative period and implement targeted preventive interventions.

**Table 5:** Confusion matrix analysis.

AUC (95%CI)	Accuracy (95%CI)	Sensitivity (95%CI)	Specificity (95%CI)	PPV (95%CI)	NPV (95%CI)	Cut off
0.89 (0.85–0.94)	0.84 (0.79–0.89)	0.77 (0.68–0.87)	0.88 (0.82–0.93)	0.76 (0.67–0.86)	0.88 (0.83–0.94)	0.38

### Internal validation

To assess the robustness of the prediction model and account for potential overfitting, we performed an internal validation using 500 bootstrap resamples following the TRIPOD guidelines (Table 6). The bootstrap-corrected AUC was 0.886 (95% CI: 0.841–0.931), compared with the apparent AUC of 0.893, yielding an optimism of 0.006. The calibration slope was 1.000 after correction (ideal value: 1.0), and the calibration intercept was < 0.001, indicating excellent agreement between predicted probabilities and observed outcomes. These findings confirm that the nomogram has good discriminative ability and calibration without significant overfitting, supporting its clinical utility for early risk stratification.

**Table 6:** Bootstrap internal validation metrics for the prediction model.

Metric	Definition	Estimate	Optimism	Corrected estimate
AUC (C-index)	Discriminative ability	0.893	0.006	0.886
Calibration slope	Agreement between predicted and observed risk	1.000	< 0.001	1.000
Calibration intercept	Calibration-in-the-large	< 0.001	–	< 0.001

Optimism represents the overestimation bias due to model overfitting, estimated via 500 bootstrap resamples. An ideal calibration slope is 1.0; an ideal intercept is 0.

## Discussion

GHF is one of the common serious traumatic injuries in the elderly and its incidence is increasing with the ageing population (3). The mechanisms and influencing factors of postoperative kinesiophobia, an important factor hindering the early recovery of patients, have not been fully elucidated (25). In this study, we analysed 209 GHF patients in the early postoperative period and found that the kinesiophobia group had significantly longer operative times, higher estimated blood loss, higher VAS pain scores, older age, and lower HHS scores compared to the non-kinesiophobia group. Multivariate analysis identified operative time, estimated blood loss, VAS score, HHS score, and age as independent predictors of early postoperative kinesiophobia. A nomogram risk model incorporating these five concurrently assessed factors demonstrated good discriminative ability with an AUC of 0.89, providing a new tool for the early clinical identification of patients with elevated fear levels in the immediate postoperative period.

First, this study found that early postoperative kinesiophobia after GHF was positively correlated with age, with patients in the kinesiophobia group being significantly older, which is closely related to physiological decline due to aging (26). Hjelholt et al. found through a large cohort study that age is an important predictor of postoperative prognosis in GHF patients (27). As patients advance in age, they experience progressive declines in muscle strength, joint flexibility, and physiological reserve, which may heighten their fear of exercise-induced pain and re-injury (28). Moreover, elderly patients tend to have comorbid chronic diseases, which further undermines confidence in recovery. Wang et al. reported that among fracture patients, older patients tended to show lower compliance with postoperative rehabilitation training, which further confirmed the association between age and kinesiophobia (29). Pain is the most central influence on kinesiophobia, and the results of this study showed that for every 1-point increase in pain score, the risk of kinesiophobia increased by 3.57 times. This finding is consistent with previous studies. Xu et al. reported through a retrospective study that the higher the degree of pain in GHF patients, the higher the risk of developing motor phobia disorder after surgery (22). Furthermore, Li et al. also found through the observation of 245 fracture patients that the pain level was an important influencing factor for the occurrence of kinesiophobia in patients (30). At the level of physiological mechanisms, pain creates a vicious cycle of ‘pain-fear-activity avoidance’ by activating fear circuits in the limbic system of the brain, especially the amygdala and anterior cingulate cortex overreactions (31). Acute postoperative pain, as an injurious stimulus, reinforces patients’ conditioned reflexes to exercise-induced pain, and this learned fear persists even when tissue damage has been gradually repaired (32). Neuroimaging studies confirmed the existence of functional reorganisation of fear-related brain regions in patients with chronic pain, which is consistent with the significant role of pain in affecting kinesiophobia in the present study, suggesting that altered neuroplasticity of pain perception may be the key mechanism (33). Therefore, in the early postoperative period, healthcare professionals may consider actively take measures to control patients’ pain, such as the rational use of analgesic drugs and psychological counselling, in order to reduce patients’ pain perception and lower the risk of kinesiophobia.

Furthermore, this study identified operative time and estimated blood loss as independent surgical predictors of kinesiophobia. Multivariate analysis confirmed that each 10-minute increase in operative time was associated with a 1.75-fold increase in kinesiophobia risk, and each 10-mL increase in estimated blood loss was associated with an 18% increase in risk. These findings suggest that greater surgical invasiveness may contribute to the development of kinesiophobia. Prolonged operative time and increased blood loss often indicate more complex fractures or more extensive surgical dissection, which can lead to heightened postoperative inflammation, more severe pain, and delayed functional recovery (34, 35). Patients who experience greater intraoperative trauma may perceive their physical condition as more fragile, thereby intensifying their fear of movement and re-injury. From a clinical perspective, these results highlight the importance of minimizing surgical invasiveness where possible. For patients with anticipated longer operative times or greater blood loss, surgeons may consider consider enhanced perioperative pain management protocols and early psychological preparation to mitigate the risk of kinesiophobia (36, 37). It should also be noted that the relationship between fracture type, surgical procedure, and our study variables was clinically heterogeneous. In our cohort, 17 of 73 patients with trochanteric fractures underwent arthroplasty rather than internal fixation—a reasonable choice for elderly patients with unstable fractures and poor bone quality, which may permit earlier full weight-bearing despite potentially greater blood loss compared with PFNA. Conversely, some patients with femoral neck fractures underwent internal fixation, which generally involves shorter operative time and less blood loss than hemiarthroplasty but may limit early unrestricted weight-bearing. These differences could theoretically influence operative time, blood loss, early functional status, and ultimately kinesiophobia. However, neither fracture type nor surgical method differed significantly between the kinesiophobia and non-kinesiophobia groups in univariate analysis (Table 2, both *P* > 0.05), and neither entered the multivariate model, suggesting that the observed associations are likely driven by overall surgical invasiveness rather than fracture type or procedure *per se*. Nevertheless, the relatively small number of patients in specific fracture–procedure subgroups precluded granular subgroup analysis, and future studies with larger sample sizes are warranted. In this study, the HHS score was significantly negatively associated with the risk of kinesiophobia, and this association can be explained by the interaction between functional status and psychological cognition. HHS score, as a comprehensive assessment index of hip function, is a direct reflection of the patients’ reduced mobility, pain tolerance, and ability to perform daily activities (38). When the HHS score is low after surgery, elderly patients tend to have a self-perception of ‘lack of exercise capacity’ due to hip dysfunction, and this sense of functional deficiency may reinforce the fear of pain or injury caused by exercise. In addition, the association between diabetes and postoperative complications and kinesiophobia also deserves attention. Metabolic disorders in patients with diabetes may affect fracture healing and pain perception (39). The occurrence of postoperative complications will increase the psychological pressure of patients. However, the independent effects of diabetes and postoperative complications were not significant in the multivariate analysis, which might be related to the sample size limit or the interaction between variables. An alternative explanation is that these factors may influence kinesiophobia indirectly through their effects on pain perception and functional recovery, with VAS and HHS scores potentially serving as mediating variables in the causal pathway. To exclude multicollinearity as a competing explanation, we performed a *post-hoc* VIF analysis, which showed all VIF values below 2.0, indicating that collinearity was not a major concern in our model. Nevertheless, given the exploratory nature of this *post-hoc* interpretation, the sample size needs to be further expanded for verification in the future.

This study constructed a nomogram risk model by integrating five factors concurrently associated with early postoperative kinesiophobia: operative time, estimated blood loss, VAS score, HHS score, and age. It is important to emphasize that, because the VAS and HHS scores were assessed simultaneously with the TSK on postoperative day 5, this model does not provide true prospective prediction of a future outcome. Rather, it serves as a risk stratification tool based on factors measurable in the immediate postoperative period that are associated with concurrent fear levels. The model demonstrated good discriminative ability and clinical utility, as verified by ROC curves (AUC = 0.89), calibration curves, and decision curve analysis. Internal bootstrap validation further confirmed the model's robustness, with a corrected AUC of 0.886 and a minimal optimism of 0.006, indicating no significant overfitting. From a surgical perspective, this nomogram provides a practical bedside tool for orthopaedic surgeons to identify patients with elevated concurrent fear levels in the immediate postoperative period. With the optimal risk threshold of 0.38 (derived from the Youden index), patients with a model-assigned probability ≥ 0.38 are classified as high-risk. For such patients, surgeons can consider targeted perioperative interventions. Conversely, low-risk patients (probability < 0.38) can follow standard rehabilitation pathways, thereby optimizing the allocation of medical resources. By integrating easily available intraoperative and early postoperative variables, this nomogram supports personalized, surgery-guided rehabilitation planning and aligns with the principles of enhanced recovery after surgery. Nevertheless, because our assessment was limited to a single early postoperative time point, we cannot determine whether the observed kinesiophobia represents a transient phenomenon that will resolve with pain control and improved mobility, or a persistent condition that will interfere with long-term rehabilitation and functional recovery.

Despite the valuable findings of this study, several limitations should be acknowledged. First, the single-time-point, cross-sectional design (postoperative day 5) precludes determination of causality and cannot distinguish transient early postoperative fear from persistent kinesiophobia that may interfere with long-term rehabilitation. Future longitudinal studies with multiple follow-up time points (e.g., 1, 3, and 6 months) are needed to characterize the trajectory of kinesiophobia and its long-term clinical relevance. Second, the fixed order of questionnaire administration (VAS first, then HHS, and finally TSK) may have introduced fatigue effects that influenced responses to the later assessments, particularly the 17-item TSK. Third, the TSK is a subjective self-assessment scale that may be affected by patients’ cognitive status; future studies could complement it with objective behavioral indicators. Fourth, several potential confounders—including fracture complexity (e.g., comminution, displacement), surgeon experience, and specific implant types (e.g., PFNA versus DHS, hemiarthroplasty versus total hip arthroplasty)—were not captured. Although anesthesia method and broad surgical procedure categories did not differ significantly between groups (Table 2), these more granular variables could influence surgical invasiveness, postoperative recovery, and kinesiophobia, and should be collected in future larger-scale studies. Fifth, the HHS was originally developed for outpatient follow-up; its functional items relied on patients’ recall of pre-fracture status, and hip range of motion assessment in arthroplasty patients was restricted by early postoperative precautions (e.g., hip flexion ≤ 90°). Future studies should consider instruments specifically validated for acute inpatient assessment, such as the Barthel Index or Functional Independence Measure (FIM).

## Conclusion

The aim of this study was to investigate the current status and surgery-related influencing factors of early postoperative kinesiophobia in GHF patients. The results showed that the incidence of kinesiophobia at postoperative day 5 was 34.0%, and operative time, estimated blood loss, VAS score, HHS score, and age were identified as factors independently associated with concurrent fear levels. The nomogram risk model constructed based on these five factors demonstrated good discriminative ability (AUC = 0.89) and may assist clinicians in the early identification of patients with elevated early postoperative fear. However, given the cross-sectional, single-time-point design of this study, it is not possible to determine whether this early fear represents a transient phenomenon or a persistent condition that will affect long-term rehabilitation outcomes. This study provides a basis for the early identification and monitoring of early postoperative kinesiophobia in GHF patients. From a surgical perspective, clinical interventions could consider minimizing surgical invasiveness where possible, implementing effective multimodal analgesia protocols, and promoting early functional recovery to reduce early fear levels. Future longitudinal studies are needed to evaluate the trajectory of kinesiophobia and to establish whether early risk stratification can improve long-term functional outcomes.

## Data Availability

The raw data supporting the conclusions of this article will be made available by the authors, without undue reservation.
